# Supplementary material for: The basis of easy controllability in Boolean networks
Source: Nat Commun. 2021 Sep 1;12:5227. doi: 10.1038/s41467-021-25533-3 (PMC8410781; doi:10.1038/s41467-021-25533-3)
Supplement: Supplementary file 1 — Supplementary Information [file 41467_2021_25533_MOESM1_ESM.pdf]

Supplementary material for: The basis of easy controllability in Boolean networks

Enrico Borriello, Bryan C. Daniels

The effects of asynchronous updating

In our study, we assume dynamics in which nodes are updated deterministically, and in a synchronous way. Although this allows for much more straightforward analysis, it is known that more realistic, asynchronous updating schemes create important differences in the types of dynamics displayed by Boolean networks [1, 2, 3, 4]. In this section, we detail those differences and explore how they affect our control analysis.

An asynchronous update rule reduces the total number of attractors from r in the synchronous case to a smaller r'. Fixed-points are preserved, while the number of cycles is reduced. Before proceeding any further it is important to stress that our logarithmic scaling result stands even when excluding all networks that have any cycles, leaving 24 of the 49 networks that we specifically highlight in Fig. S2F. Furthermore, we do not have a reason to believe that an asynchronous updating scheme would fundamentally change the result for the networks we test. This is because any loss of cyclic attractors in the asynchronous case would cause a corresponding decrease in the size of witness sets and therefore CKs.

More specifically, cycles are not only reduced, but their definition is now made less precise. When node states are updated with a random period, the network no longer transitions through an ordered cycle of states, and basins are now a more useful concept. Distinct cycles correspond now to distinct basins, i.e. regions B_k of the configuration space such that the transition probability between states $\mathbf{X}_i$ in B_i and $\mathbf{X}_j$ in B_j is equal to zero if $i \neq j$.

Let us now consider the dynamics within one of these basins. If all nodes keep changing for $t \to \infty$, then this "non-constant attractor" is not controllable. In that case, our definition of control kernel becomes irrelevant as it only accounts for static control. Therefore, we can consider cases where at least some nodes within the basin converge to fixed values, while the remaining nodes have non-constant values. Analogously to Ref. [5], we will refer to this pseudo-Boolean vector where some entries remain unassigned as a 'quasi-attractor' of this basin. The quasi-attractor of a fixed point is clearly the fixed-point itself.

We can now proceed in a similar way to the synchronous case.

Distinguishing a fixed-point attractor: The witness sets of these vectors will each be the same size or smaller than their counterparts in the case where all r' vectors have all their entries assigned. (This is analogous to distinguishing fixed-points in the synchronous case when considering the additional freedom granted by the cycling components.) The asynchronous CK for a fixed point will never be greater than the size of the synchronous CK.

Distinguishing a "non-constant" attractor: We can proceed in the same way we did with synchronous cyclic attractors. For each quasi-attractor, we restrict the analysis to the n' columns where all values are constant. This corresponds to a minimal witness set problem with r' attractors and n' bits per vector. (Notice that some entries in the remaining vectors might still be non-constant, but this does not increase the size of the witness set.) We know that the reduced n' does not have a direct effect on the size of the witness set. Though it is the case that strings of reduced length are more likely to coincide, if the reduced string of our attractor has a duplicate in the set, then the non-constant attractor cannot be controlled (as in the analysis of synchronous cycles).

In this case, then, we expect a similar result for the size of witness sets and therefore control kernels: Given the new number r' of quasi-attractors, we expect to be able to approximate the control kernel by solving a minimal witness set problem with r' vectors, and we therefore expect $\langle |CK| \rangle \sim \log_2 r'$.

Relation between controllability and other network attributes

In the main text, we argue that the logarithmic scaling of CK sizes might be expected for networks that have many input nodes or otherwise consist of many small hierarchical modules. This motivates checking whether networks with few input nodes or large modules still obey the scaling. As shown in Fig. S2 C and D, even restricting the analysis to these more "difficult" networks preserves our main result.

Additionally, Fig. S2 A and B indicate the properties of networks in the Cell Collective database that we are unable to analyze due to the computational complexity of identifying all attractors and CKs. Finally, in Fig. S2 E and F, we highlight average control kernel sizes for those networks that do and do not have cyclic attractors (see above supplemental section for a discussion on the effects of asynchronous updating on cyclic attractors).

"Smallest perturbations" of the projective plane construction

We have seen that sets of binary vectors with $\langle |w| \rangle > \log_2 r$ are possible, and know examples can be built exploiting the geometric properties of "large" finite projective planes. We now want to start exploring how likely these sets of vectors are by considering the smallest perturbations of the projective plane counterexample, i.e. the effect of flipping just one entry in one of its vectors. There are four possible cases to consider:

1. $0 \to 1$ in a point

2. $1 \to 0$ in a point
3. $0 \to 1$ in a line
4. $1 \to 0$ in a line

$0 \to 1$ in a point ("P01 case")

Let us call **P** the point hosting the flip, and **Q** the point identified by a 1 in the coordinate where the flip happened. The new attractor, **A**, is now a vector with two 1s.

Only one line has two 1s in those same coordinates, let us call it $\mathbf{L}_{\mathrm{PQ}}$. We can pin them to 1, and a coordinate corresponding to a different point than **P** and **Q** on $\mathbf{L}_{\mathrm{PQ}}$ to 0. This is enough to identify **A**, so $w_{\mathrm{A}} = 3$.

Let us check that this does not increase w_{B}, for any other attractor **B**. $\mathbf{L}_{\mathrm{PQ}}$ can be identified equally well by a different pair of 1s. Therefore, its w is still 2. The other lines are not affected. Among the other points, only **Q** is affected by the flip. To identify **Q**, pinning its 1 value is not enough. We also need to pin coordinates to 0 to exclude the $p+1$ lines on it. In particular, we need to exclude $\mathbf{L}_{\mathrm{PQ}}$. In doing so, we can pin the characteristic coordinate of **P** to zero. This will also distinguish **Q** from **A**.

Therefore, a $0 \to 1$ flip in a point only affects the minimal number of distinguishing nodes of that attractor, and reduces it from $p+2$ to 3. If we call $\langle |w_{PP}| \rangle$ the average of the unperturbed case, eq. (6) in the main text, and $\langle |w_{P01}| \rangle$ the new average,

$$\langle |w_{PP}| \rangle - \langle |w_{P01}| \rangle = \frac{p+2}{2q} - \frac{3}{2q} = \frac{p-1}{2q} \ . \tag{1}$$

With $p \geq 16$, this difference is less than 0.03. A single flip of this kind is not enough to bring this configuration below the $\log_2 r$ reference level.

$1 \to 0$ in a point ("P10 case")

This flip is replacing a point with the null vector, that we will call **A**. To distinguish **A** we need to set all coordinates, other than the one we just flipped, to 0. This also distinguishes it from all the lines. $w_{\mathrm{A}} = q - 1$. Besides, this flip does not affect any other vector, as we always need to set at least one coordinate do 1 to distinguish either a point or a line, and this automatically excludes **A**.

$$\langle |w_{PP}| \rangle - \langle |w_{P10}| \rangle = \frac{p+2}{2q} - \frac{q-1}{2q} = -\frac{p^2-2}{2q} \ . \tag{2}$$

This flip is actually *increasing* $\langle |w| \rangle$ even further. For $p = 17$, this change is still relatively small, and equal to 0.47, to be compared to $\log_2 614 = 9.16$ and $\langle |w_{PP}| \rangle|_{r=614} = 10.5$ ($p = 13$ gives rise to $r = 366$, which is very close to threshold.).

$0 \to 1$ in a line

Let's call $\mathbf{P}_i, \ldots, \mathbf{P}_{p+1}$ the points on the line hosting the flip. We now flip the characteristic coordinate of point $\mathbf{Q}$ to 1 and call this new attractor $\mathbf{A}$. We can always choose to set the characteristic coordinates of $\mathbf{Q}$ to 0 as part of our procedure do distinguish $\mathbf{P}_k$ from $\mathbf{L}_{\mathrm{P}_k\mathrm{Q}}$. This automatically excludes $\mathbf{A}$. Therefore, we can reduce w_k from $p+2$ to $p+1$ for all $\mathbf{P}_k$.

On the contrary, this does not affect w_j for j corresponding to the other lines. Lines are identified by two points, and we can always exclude $\mathbf{Q}$ in choosing those points. We can do the same with $\mathbf{A}$.

$$\langle |w_{PP}| \rangle - \langle |w_{L01}| \rangle = \frac{(p+1)(p+2)}{2q} - \frac{(p+1)^2}{2q} = \frac{p+1}{2q} \ . \quad (3)$$

This is less than 0.002 with $r = 614$.

$1 \to 0$ in a line

Let us call $\mathbf{P}$ the point identified by the coordinate we flip, and $\mathbf{A}$ the new attractor we obtain. Before the flip, $\mathbf{A}$ was a line containing $\mathbf{P}$. Now we need to distinguish $\mathbf{P}$ from one less line. No other attractor is affected.

$$\langle |w_{PP}| \rangle - \langle |w_{L10}| \rangle = \frac{p+2}{2q} - \frac{p-1}{2q} = \frac{1}{2q} \ . \quad (4)$$

With $r = 614$, this difference is a negligible 0.07.
These considerations show that this projective plane counterexample is stable to the smallest changes to its initial configuration. But, it is important to notice that these perturbations do not really affect the strong bias of zeroes to ones. When these vectors are interpreted as attractor states, the relevance of sets with such a strong bias in modeling real world system remains dubious.

These examples are compared to $\log_2 r$ in Fig. S3. Notice that the increase in $\langle |w| \rangle$ due to a P10 perturbation, eq. (2), also reduces the minimal order p for which $\langle |w| \rangle > \log_2 r$ from 17 to 13, and r from 614 to 366.

Control kernels and network circuits

Figure S5 shows the overlap among sets of controlling nodes using alternative methods, as discussed in the main text.

Validating attractors

Our results depend crucially on correctly identifying the number of attractors in each network. In cases of relatively small networks, it is possible to check the results of our modular approach with a more direct approach that simply finds all closed loops in the explicitly enumerated state transition graph. For all such cases, consisting of 22 biological and 375

random networks, we have checked that the number of attractors matches exactly using the two techniques. In addition, we have checked that the biological cases analyzed using stable motif analysis have the same number of attractors in the 15 cases of networks we were able to analyze that have only fixed point attractors (where the definitions of our attractors and stable motif's quasi-attractors match).

Outlier random networks

Attractors, basin sizes and control kernel sizes of the eight outlier, random threshold networks highlighted in Fig. 9A in the main text. These networks share a bias toward excitation that creates repellor fixed points.

Outlier 1

**attractor**	**basin size**	$\|CK\|$
1 1 0 1 1 1 0 0 1 1	600	1
1 1 0 1 1 1 0 0 1 0	422	2
0 0 0 0 0 1 0 0 0 0	1	8
0 0 0 0 0 0 0 0 0 0	1	10

Outlier 2

**attractor**	**basin size**	$\|CK\|$
1 1 1 1 1 1 1 1 1 1	1023	1
0 0 0 0 0 0 0 0 0 0	1	10

Outlier 3

attractor	basin size	$\lvert CK \rvert$
1 1 1 1 1 1 1 0 1 1 1 1 1 0 1	16384	1
1 1 1 1 1 1 1 0 1 1 0 1 1 0 1	8192	2
1 1 1 1 1 1 1 0 1 1 0 1 0 0 1	4088	4
1 1 1 1 1 1 1 0 1 1 0 0 0 0 1	4088	4
0 0 0 0 0 0 0 0 0 0 0 0 0 0 1	1	14
0 0 0 1 1 0 0 0 0 0 0 1 0 0 1	1	14
0 0 0 1 1 0 0 0 0 0 0 1 0 0 0	1	15
0 0 0 1 1 0 0 0 0 0 0 0 0 0 1	1	14
0 0 0 0 0 0 0 0 0 0 0 1 0 0 1	1	14
0 0 0 1 0 0 0 0 0 0 0 1 0 0 1	1	14
0 0 0 1 0 0 0 0 0 0 0 0 0 0 1	1	14
0 0 0 0 1 0 0 0 0 0 0 1 0 0 1	1	14
0 0 0 0 1 0 0 0 0 0 0 0 0 0 1	1	14
0 0 0 1 0 0 0 0 0 0 0 1 0 0 0	1	15
0 0 0 1 0 0 0 0 0 0 0 0 0 0 0	1	15
0 0 0 0 1 0 0 0 0 0 0 1 0 0 0	1	15
0 0 0 0 1 0 0 0 0 0 0 0 0 0 0	1	15
0 0 0 0 0 0 0 0 0 0 0 1 0 0 0	1	15
0 0 0 1 1 0 0 0 0 0 0 0 0 0 0	1	15
0 0 0 0 0 0 0 0 0 0 0 0 0 0 0	1	15

Outlier 4

attractor	basin size	$\lvert CK \rvert$
1 1 1 1 1 1 1 1 1 1 1 1 1 1 1	32767	1
0 0 0 0 0 0 0 0 0 0 0 0 0 0 0	1	15

Outlier 5

attractor	basin size	$\lvert CK \rvert$
1 1 1 1 1 1 1 1 1 1 1 1 1 1 1	32766	1
0 1 0 0 0 0 0 0 0 0 0 0 0 0 0	1	15
0 0 0 0 0 0 0 0 0 0 0 0 0 0 0	1	15

Outlier 6

attractor	basin size	$\lvert CK \rvert$
1 1 1 1 1 1 1 1 1 1 1 1 1 1 1	32767	1
0 0 0 0 0 0 0 0 0 0 0 0 0 0 0	1	15

Outlier 7

**attractor**	**basin size**	$\|CK\|$
1 1 1 1 0 0 1 1 1 1 1 1 1 1 1	23054	1
1 0 1 1 0 0 1 1 1 1 1 1 1 1 1	5630	2
0 0 1 1 0 0 1 1 1 1 1 1 1 1 1	4083	4
0 0 0 0 0 0 0 0 0 0 0 0 0 0 0	1	15

Outlier 8

**attractor**	**basin size**	$\|CK\|$
0 1 1 1 1 1 1 1 1 1 1 1 1 0 1 1 1 1 1 1	358134	2
0 1 1 1 1 1 1 1 1 1 1 1 1 0 0 1 1 1 1 1	277682	3
1 1 1 1 1 1 1 1 1 1 1 1 1 0 0 1 1 1 1 1	243000	2
1 1 1 1 1 1 1 1 1 1 1 1 1 0 1 1 1 1 1 1	169758	2
0 0 0 0 0 0 0 0 1 0 0 0 0 0 0 0 0 0 0 0	1	20
0 0 0 0 0 0 0 0 0 0 0 0 0 0 0 0 0 0 0 0	1	20

References

[1] Inman Harvey and Terry Bossomaier. Time out of joint: Attractors in asynchronous random boolean networks. *Proceedings of the Fourth European Conference on Artificial Life*, pages 67–75, 1997.

[2] Florian Greil and Barbara Drossel. Dynamics of critical Kauffman networks under asynchronous stochastic update. *Physical Review Letters*, 95(4):3–6, 2005.

[3] Konstantin Klemm and Stefan Bornholdt. Stable and unstable attractors in Boolean networks. *Physical Review E - Statistical, Nonlinear, and Soft Matter Physics*, 72(5):1–4, 2005.

[4] Jordan C. Rozum, Jorge Gomez Tejeda Zañudo, Xiao Gan, and Réka Albert. Parity and time-reversal elucidate decisions in high-dimensional state space - application to attractor scaling in critical boolean networks. *arXiv*, 2009.05526, 2020.

[5] Jorge G.T. Zañudo and Réka Albert. Cell Fate Reprogramming by Control of Intracellular Network Dynamics. *PLoS Computational Biology*, 11(4):1–24, 2015.

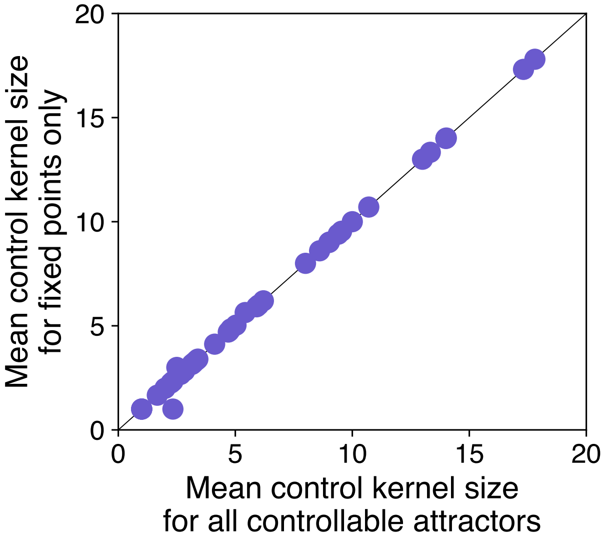

Figure S1: Mean control kernel sizes are not strongly affected when restricting the average to fixed point attractors.

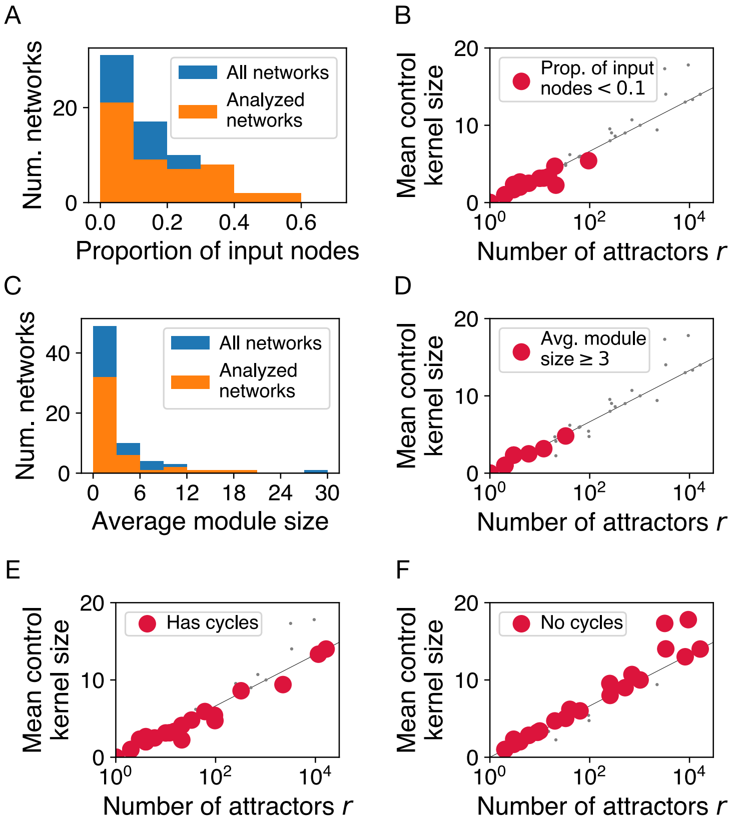

Figure S2: We may expect networks with many input nodes and small modules to have control kernels with size roughly $\log_2 r$. But even restricting to those with few input nodes and large modules (red points in B and D), the analyzed networks closely follow this logarithmic scaling. A and C: the number of networks with given network statistics among all networks in the Cell Collective database (blue) and the 49 for which we were able to successfully find all control kernels (orange). E and F: Mean control kernel sizes highlighting networks that have cyclic attractors and those that do not.

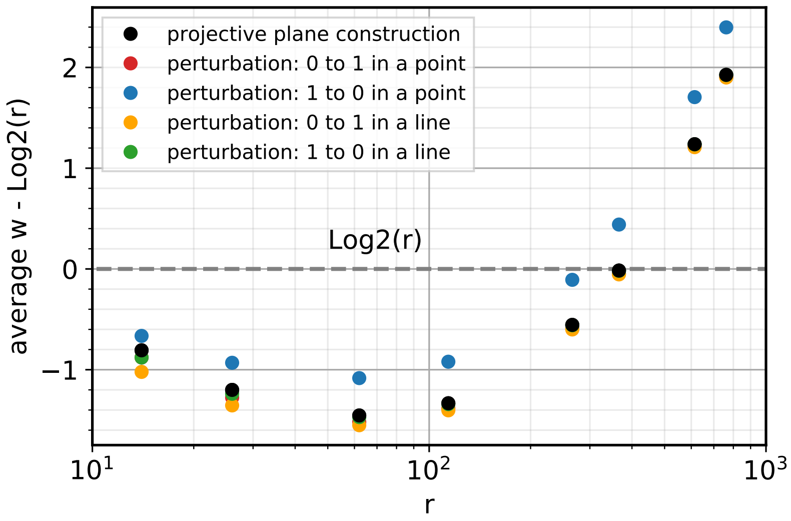

Figure S3: Perturbations of the projective plane construction.

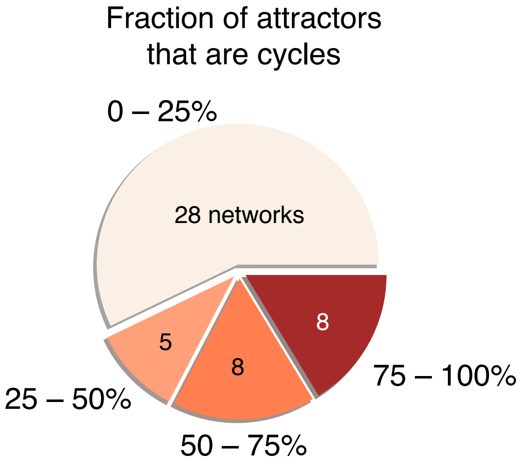

Figure S4: Many of the analyzed biological networks have relatively few cyclic attractors.

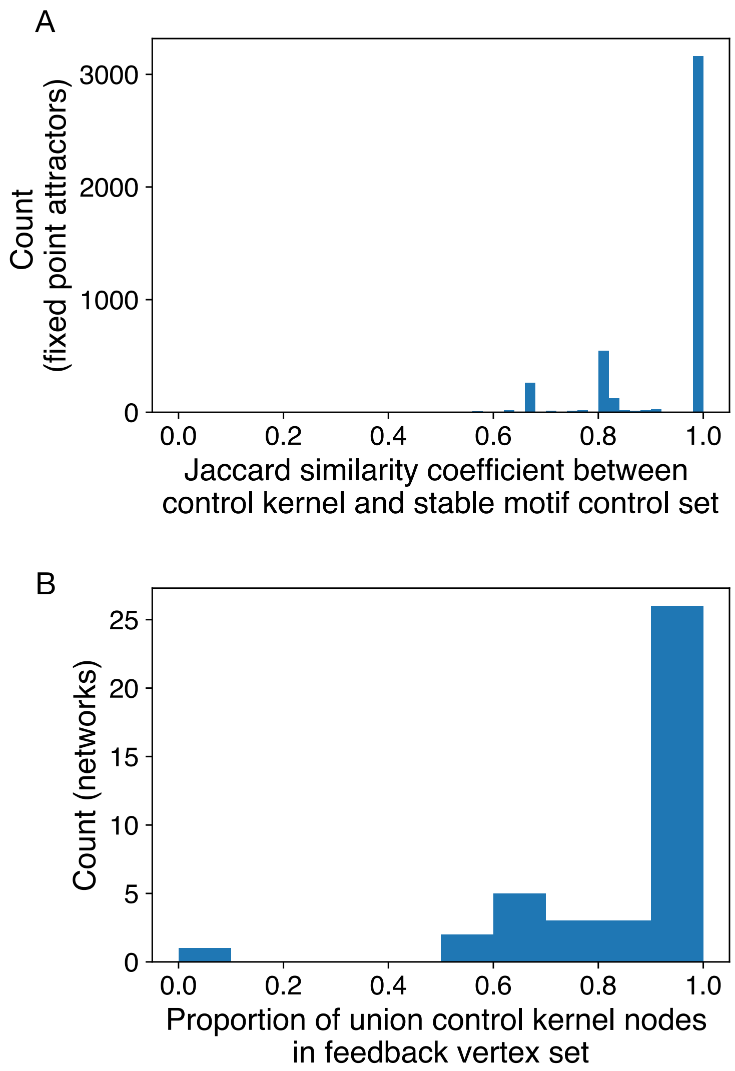

Figure S5: Comparison between our control kernel results for the biological networks with those obtained using two alternative methods for computing controlling nodes: (A) stable motifs and (B) feedback vertex sets. In the latter case, while the average overlap is still high, smaller values in some cases are explained by the additional ambiguity affecting our union sets: unions of minimal size sets are not necessarily minimal size control kernel sets for *all* the attractors.

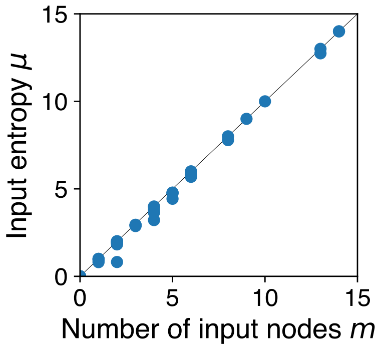

Figure S6: The "input entropy" μ is typically close to the number of input nodes m in the 49 biological networks we test.

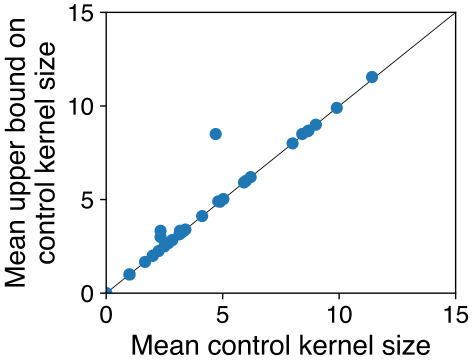

Figure S7: The upper bound on control kernel size in the main text's Eq. 2 is often tight. Here control kernels and the iterative bound on control kernels were computed for 40 networks using the sampling method.
