# Supplementary material for: The basis of easy controllability in Boolean networks
Source: Nat Commun. 2021 Sep 1;12:5227. doi: 10.1038/s41467-021-25533-3 (PMC8410781; doi:10.1038/s41467-021-25533-3)
Supplement: Supplementary file 3 — Reporting Summary [file 41467_2021_25533_MOESM3_ESM.pdf]

nature portfolio

Corresponding author(s): Enrico Borriello and Bryan Daniels

Last updated by author(s): Aug 10, 2021

Reporting Summary

Nature Portfolio wishes to improve the reproducibility of the work that we publish. This form provides structure for consistency and transparency in reporting. For further information on Nature Portfolio policies, see our Editorial Policies and the Editorial Policy Checklist.

Statistics

For all statistical analyses, confirm that the following items are present in the figure legend, table legend, main text, or Methods section.

n/a	Confirmed	
☐	☒	The exact sample size (n) for each experimental group/condition, given as a discrete number and unit of measurement
☐	☒	A statement on whether measurements were taken from distinct samples or whether the same sample was measured repeatedly
☒	☐	The statistical test(s) used AND whether they are one- or two-sided<br>*Only common tests should be described solely by name; describe more complex techniques in the Methods section.*
☒	☐	A description of all covariates tested
☒	☐	A description of any assumptions or corrections, such as tests of normality and adjustment for multiple comparisons
☐	☒	A full description of the statistical parameters including central tendency (e.g. means) or other basic estimates (e.g. regression coefficient) AND variation (e.g. standard deviation) or associated estimates of uncertainty (e.g. confidence intervals)
☒	☐	For null hypothesis testing, the test statistic (e.g. F, t, r) with confidence intervals, effect sizes, degrees of freedom and P value noted<br>*Give P values as exact values whenever suitable.*
☒	☐	For Bayesian analysis, information on the choice of priors and Markov chain Monte Carlo settings
☒	☐	For hierarchical and complex designs, identification of the appropriate level for tests and full reporting of outcomes
☒	☐	Estimates of effect sizes (e.g. Cohen's d, Pearson's r), indicating how they were calculated

Our web collection on statistics for biologists contains articles on many of the points above.

Software and code

Policy information about availability of computer code

Data collection	No software was used for data collection.
Data analysis	The python code used to calculate control kernels is available as a Zenodo repository under the accession code 10.5281/zenodo.5172898 [https://doi.org/10.5281/zenodo.5172898]. This code depends on a number of open-source software packages: neet, datadex, numpy, and networkx.

For manuscripts utilizing custom algorithms or software that are central to the research but not yet described in published literature, software must be made available to editors and reviewers. We strongly encourage code deposition in a community repository (e.g. GitHub). See the Nature Portfolio guidelines for submitting code & software for further information.

Data

Policy information about availability of data

All manuscripts must include a data availability statement. This statement should provide the following information, where applicable:

- Accession codes, unique identifiers, or web links for publicly available datasets
- A description of any restrictions on data availability
- For clinical datasets or third party data, please ensure that the statement adheres to our policy

The control kernel size data generated in this study have been deposited in as a Zenodo repository under the accession code 10.5281/zenodo.5172898 [https://doi.org/10.5281/zenodo.5172898].

The biological network models analyzed in this study are available on the Cell Collective website [https://cellcollective.org/].

Field-specific reporting

Please select the one below that is the best fit for your research. If you are not sure, read the appropriate sections before making your selection.

- [x] Life sciences
- [ ] Behavioural & social sciences
- [ ] Ecological, evolutionary & environmental sciences

For a reference copy of the document with all sections, see nature.com/documents/nr-reporting-summary-flat.pdf

Life sciences study design

All studies must disclose on these points even when the disclosure is negative.

Sample size	We use a convenience sample of all 49 biological network models that are in an existing database and can be analyzed in a reasonable amount of time by our method. This is sufficient to justify our mild conclusion that "biological networks may be typically easy to control". Our main conclusions regard the mathematical basis of this easy controllability, which does not rely on sampling.
Data exclusions	Networks were excluded from analysis when we were unable to analyze control kernels for all attractors using 2 days of CPU time. We compare network statistics for these networks to the successfully analyzed networks in Supplemental Figure S2.
Replication	In the few cases (5 of 49) in which we use the sampling approach for estimating control kernel sizes, conclusions were successfully verified by increasing the number of random samples by a factor of 10. All other calculations are mathematically deterministic, so no replication is necessary.
Randomization	Attempting to get a random sample across all types of biological regulatory networks is beyond the scope of this study. Instead, we use a convenience sample of 49 existing network models that are analyzable using our method. Our main conclusions involve the mathematical basis of control kernel sizes, which is a separate question from whether small control kernel sizes are seen more generally in biology.
Blinding	Blinding is not relevant to this study because all results are computed algorithmically, without human intervention.

Reporting for specific materials, systems and methods

We require information from authors about some types of materials, experimental systems and methods used in many studies. Here, indicate whether each material, system or method listed is relevant to your study. If you are not sure if a list item applies to your research, read the appropriate section before selecting a response.

Materials & experimental systems

n/a	Involved in the study	
[x]	[ ]	Antibodies
[x]	[ ]	Eukaryotic cell lines
[x]	[ ]	Palaeontology and archaeology
[x]	[ ]	Animals and other organisms
[x]	[ ]	Human research participants
[x]	[ ]	Clinical data
[x]	[ ]	Dual use research of concern

Methods

n/a	Involved in the study	
[x]	[ ]	ChIP-seq
[x]	[ ]	Flow cytometry
[x]	[ ]	MRI-based neuroimaging
